# The association between cystatin C and COPD: a meta-analysis and systematic review

**DOI:** 10.1186/s12890-020-01208-5

**Published:** 2020-06-26

**Authors:** Limin Chai, Wei Feng, Cui Zhai, Wenhua Shi, Jian Wang, Xin Yan, Qingting Wang, Qianqian Zhang, Manxiang Li

**Affiliations:** Department of Respiratory and Critical Care Medicine, the First Affiliated Hospital of Xi’an Jiaotong University, No. 277, West Yanta Road, Xi’an, 710061 Shaanxi People’s Republic of China

**Keywords:** Cys C, COPD, Exacerbation, Meta-analysis

## Abstract

**Background:**

In recent years, many studies have discovered that cystatin C (Cys C) may play an important role in respiratory diseases, especially in chronic obstructive pulmonary disease (COPD). However, the findings of these studies were inconsistent. This systematic review and meta-analysis aimed to assess the relationship between serum Cys C and COPD.

**Methods:**

We conducted a systematic literature search in PubMed, Embase, Web of Science, Wanfang databases, and the China National Knowledge Infrastructure. The standardized mean difference (SMD), Fisher’s Z-value and 95% confidence interval (CI) were calculated to investigate the effect sizes. Subgroup analyses were performed on disease status, ethnicity, assay method, and study design. Sensitivity was performed, and publication bias was assessed.

**Results:**

A total of 15 studies, including 4079 COPD patients and 5949 controls, were included in this meta-analysis. The results showed that serum Cys C levels in patients with COPD were significantly higher than those in controls (SMD = 0.99, 95% CI =0.62–1.37, *P* < 0.001), especially in AECOPD (SMD = 1.59, 95% CI =1.05–2.13, *P* < 0.001), and there were statistically different among AECOPD and SCOPD (SMD = 0.35, 95% CI =0.10–0.59, *P* = 0.005). The serum Cys C levels were negatively correlated with FEV1%pre (Z = − 0.45, 95%CI = -0.58--0.32, *P* = 0.011) and FEV1/FVC (Z = − 0.32, 95%CI = -0.50--0.14, *P* = 0.006). The serum Cys C levels were independent of ethnicity, assay method, and study design.

**Conclusion:**

Serum Cys C levels were associated with COPD and COPD exacerbation, and they were inversely correlated with FEV1%pre and FEV1/FVC.

## Background

Chronic obstructive pulmonary disease (COPD) is a disease characterized by incomplete reversibility of airflow obstruction, chronic inflammation of the airways, and systemic effects or comorbidities [1–3]. Although COPD is a preventable and treatable disease, it still brings heavy economic burden to family and the society [4–6]. However, the pathogenesis of COPD is not completely elucidated, and it is the long-term interaction of many factors including environmental factors and genetic factors [7]. Nowadays, GOLD guidelines recommend using spirometry assessment as a diagnostic and prognostic indicator for COPD [8]. However, fewer than half of the patients have the data from spirometry lung function tests [9, 10]. Therefore, it is of great significance to explore simple and novel biomarkers of COPD for the early diagnosis and monitoring of prognosis.

Cystatin C (Cys C), an inhibitor of cysteine proteinase, belongs to the member of family II of the cystatin super-family [11]. Cys C is an alkaline secreted protein, with a molecular mass of 13.3 kDa, which widely exists in various tissues of nucleated cells and body fluids, and produced by the body’s all nucleated cells [12, 13]. Cys C can protect human cells from improper hydrolysis of proteases inside and outside the body. Previous researches suggest that serum Cys C is entirely determined by the glomerular filtration rate (GFR), and may more accurately estimate GFR than creatinine [14–16]. It has been suggested that Cys C is valuable in predicting cardiovascular mortality in cardiovascular disease [17, 18]. In recent years, there are many studies on the clinical significance of serum Cys C levels changes in respiratory diseases. It has been studied that Cys C plays a role in pathogenesis of obstructive sleep apnea syndrome [10–20], lung malignancy [21], pleural effusions [22], and emphysema [23].

Recent studies indicate a possible link between Cys C and COPD [24–28]; however, the conclusion is not clear. Most studies have shown that serum Cys C levels are higher in the exacerbation group than stable COPD or healthy control [29, 30], but Selder et al. [31] find that serum Cys C levels are lower in exacerbation group than stable COPD. We performed this meta-analysis to clarify the associations between serum Cys C levels and COPD as well as COPD exacerbation. This study might provide new perspectives in explaining the relationship between Cys C and COPD, COPD exacerbation.

## Methods

### Literature search strategy

We had searched PubMed, Embase, Web of Science, Wanfang databases (www.wanfangdata.com.cn), and the China National Knowledge Infrastructure (CNKI, www.cnki.net) to collect articles involving the role of Cys C in COPD, and the retrieval time was before Jul 312,019. The keywords used for searching were: (“Cystatin C” or “Cys C”) in combination with (“chronic obstructive pulmonary disease” or “COPD”). Besides, the reference lists of all selected articles were manually searched for further potentially eligible articles. There were no restrictions on language, population, publication date, or type of report, and unpublished data were excluded.

### Inclusion and exclusion criteria

All the potential studies were independently selected by two reviewers (Limin Chai and Wei Feng) based on the following inclusion and exclusion criteria. The researches in the meta-analysis accorded with the following inclusion criteria: 1) a study involving the role of Cys C in COPD; 2) cohort, case–control, or cross-section design; 3) if there was duplication of data, only the most complete and recent studies were included; and 4) the effect size and its 95% confidence interval (CI) were provided or could be estimated. *Furthermore,* the following exclusion criteria were used: 1) not related to Cys C or COPD; 2) Based on family or sibling pairs studies; 3) measured Cys C concentrations in biological samples other than blood, including urine, sputum, or BALF; 4) reviews, reports, comments, letters, meta-analysis, abstracts etc. were also excluded.

### Quality of the literature

The Newcastle-Ottawa scale (NOS) was used to assess the quality of eligible studies from three aspects: (1) selection of cases and controls; (2) comparability between cases and controls; (3) exposure in cases and controls. The NOS has a score range of zero to nine, and studies with a score of more than seven were thought to be of high quality [32].

### Data extraction

Two evaluators extracted the data independently and used a standardized form. Collected the following items: the name of the first author, year of publication, study design, country and ethnicity of participants, sample, method of Cys C measurement, sample size, serum Cys C levels, age of participant. The quality of each selected study was also independently assessed by two reviewers who used the Newcastle–Ottawa Scale. Discussion with another investigator resolved it when they had divergences.

### Statistical analysis

In this study, all the statistical analyses were carried out using STATA 12.0 software (Stata Corp LP, College Station, TX, the United States). For the continuous data, the standardized mean difference (SMD) and 95% CI were calculated. In the case of only reporting SEM, SD was estimated by the formula: SD = SEM × Sqrt (sample size) www.cochranehandbook.org.). Estimating the sample mean and standard deviation from the sample size, median, range and/or interquartile range [33]. *P* < 0.05 indicated the statistically significant. Heterogeneity among studies was assessed based on the chi-square Q test and I2 test. Heterogeneity was significant when the *P* < 0.1 or I^2^ > 50%. When there was no heterogeneity, the fixed effect model was used for analysis. Otherwise, the random effect model was used. In addition, Fisher’s r-to-Z transformation was used to convert each correlation coefficient into their approximately associated Z statistics. If the Fisher’s Z-value could not be directly obtained, which is considered the normal distribution [34]. Moreover, Begg’s tests and Egger’s tests were used to evaluating publication bias [35, 36]. Meta-regression analysis and subgroup analysis based on ethnicity, assay method, and study design were used to explore the potential sources of heterogeneity. Sensitivity analysis was applied by performing leave-one-out function to test the robustness of the pooled estimate.

## Results

### Characteristics of included studies

**Fig. 1:**
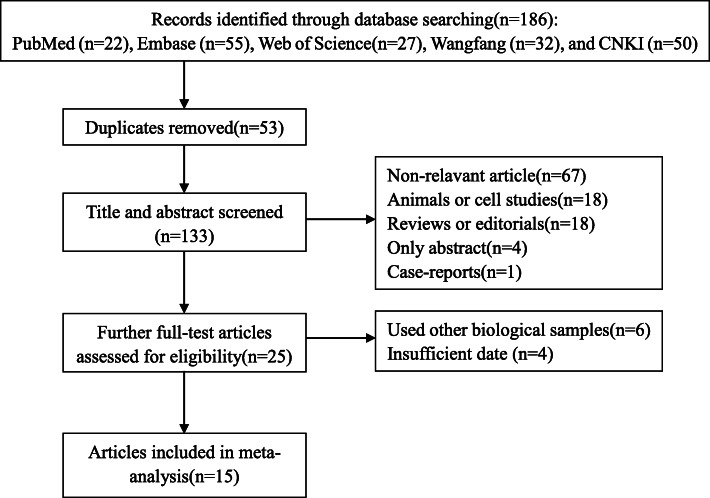
Flow chart of included/excluded studies using the criteria before the meta-analysis. Abbreviations: CNKI, China National Knowledge Infrastructure

The literature screening process and results were shown in Fig. 1. Briefly, a total of 186 articles were preliminarily distinguished from PubMed (*n* = 22), Embase (*n* = 55), web of science (*n* = 27), Wanfang (*n* = 32), and China National Knowledge Infrastructure (CNKI) (*n* = 50), among which 53 were duplicates. After screening on title and abstract, 25 articles remained for full text review. In consequence, 15 studies fulfilled the inclusion criteria, involving 4079 cases and 5949 controls. The studies, which were included in this meta-analysis, were published between 2012 and 2019 and were from the USA, Japan, Turkey, China, and Spain. The information of these studies was extracted and listed separately in the Table 1. Three articles (20%) were of moderate quality, and the other included studies (80%) were of high quality according to the NOS quality score evaluation.

**Table 1:** Characteristics of studies included in the meta-analysis Study

Author	Year	Study design	Country	Ethnicity	Sample	Sample size		Assay method	Cys C Concentration (mean±SD, mg/L)		Subjects(% men)	Age (mean±SD or median (range), year)			NOS score
						Case	Control		Case	Control		SCOPD	AECOPD	Control	
Qu et al [37]	2010	Case-control	China	Asian	serum	70	30	ELISA	SCOPD(70)1.76±0.62	0.98±0.51	62.3/NA/66.7	64.60±10.50	NA	63.50±9.20	6
Rokadia et al [38]	2012	Cross-sectional	USA	Caucasian	serum	374	5264	immunonephelometry	All(374)0.978±0.34;Emphysema(120)1.139±0.24;Chronic bronchitis(254)0.902±0.35	0.883±0.58	36.6/49.3	52.4		41.1	8
Sun et al [39]	2013	Case-control	China	Asian	serum	84	NA	immunonephelometry	SCOPD(30)0.95±0.23; AECOPD(54)1.12±0.26	NA	70.0/68.5/NA	64.17±10.03	66.98±8.07	NA	6
Zhang et al [27]	2014	Case-control	China	Asian	serum	129	122	immunonephelometry	SCOPD(129)0.115±0.05	0.105±0.03	100.0/NA/100.0	81.78± 6.68	NA	80.26±6.81	8
Zhang et al [29]	2014	Case-control	China	Asian	serum	392	151	immunonephelometry	All(392)1.04±0.21;SCOPD(299)1.03±0.21;AECOPD(93)1.09±0.22	0.84±0.18	96.1/73.1/55.0	65 (36-86)	65 (44-81)	63±6.00	8
Yoshizawa et al [40]	2015	Case-control	Japan	Asian	serum	108	73	immunonephelometry	SCOPD(108)1.2±0.4	0.9±0.2	83.3/NA/49.3	74.3±7.1	NA	71.8±7.3	9
Liu et al [41]	2015	Case-control	China	Asian	serum	132	36	ELISA	All(132)0.1287±0.0377;SCOPD(60)0.1254±0.0309;AECOPD(72)0.1315±0.0425	0.1084±0.0267	52.8/51.7/55.5	60-69	62-71	60-68	7
Zhang et al [42]	2015	Cohort	China	Asian	serum	90	90	immunonephelometry	AECOPD(90)1.31±0.30	0.87±0.13	NA/78.9/77.8	NA	63±7	62±7	7
Zhou et al [43]	2016	Case-control	China	Asian	serum	168	NA	immunonephelometry	SCOPD(60)0.94±0.22;AECOPD(108) 1.13±0.25	NA	70/67.6/NA	65.20±10.10	67.11±8.11	NA	6
Ni et al [44]	2017	Case-control	China	Asian	serum	90	45	immunonephelometry	All(90)0.1409±0.0197;SCOPD(45)0.1339±0.0154;AECOPD(45)0.1478±0.0212	0.1045±0.0209	54.4/53.3	58.1±5.4		56.9±4.9	7
Chen et al [45]	2017	Case-control	China	Asian	serum	95	20	ELISA	0.13299±0.01121	0.10617±0.02132	56.8/63.3	64.04±3.12		63.76±2.44	7
Cui et al [46]	2018	Case-control	China	Asian	serum	157	50	immunonephelometry	1.06±0.41	0.84±0.27	60.5/60	73.2±8.2		70.5 ± 4.7	7
Selda et al [31]	2018	Case-control	Turkey	Asian	serum	126	50	immunonephelometry	All(126)0.99±0.45;SCOPD(68)1.01±0.56;AECOPD(58)0.97±0.29	0.47±0.13	88.2/82.8/82.0	64.19±10.6	65.12±6.93	60.12±11.77	8
Amado et al [28]	2018	Cohort	Spain	Caucasian	serum	65	18	immunonephelometry	SCOPD(65)0.97±0.32	0.88±0.2	72/NA/55	67±7	NA	67±5	9
Dickson et al [30]	2019	Case-control	China	Asian	serum	###	NA	immunonephelometry	SCOPD(362)1.18±0.471;AECOPD(1637)1.21±0.47	NA	68.5/69.5/NA	92.97±9.83	72.51±10.27	NA	7

*Abbreviations*: *COPD* Chronic obstructive pulmonary disease, *Cys C* Cystatin C, *NOS* Newcastle-Ottawa scale, *ELISA* enzyme-linked immunosorbent assay, *AECOPD* acute exacerbation COPD, *SCOPD* stable COPD

### Serum Cys C levels in patients with COPD

**Fig. 2:**
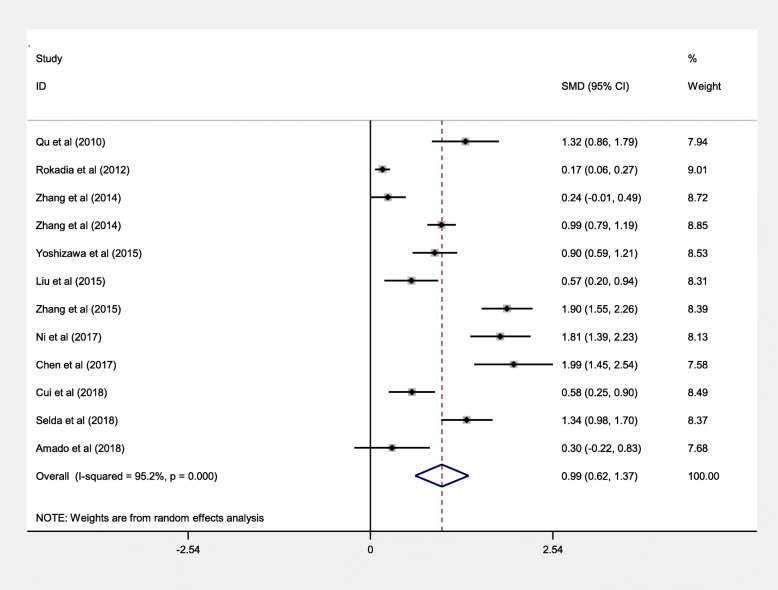
Meta-analysis of serum Cys C levels in COPD patients compared with controls. Abbreviations: SMD, standardized mean difference; CI, confidence interval

**Fig. 3:**
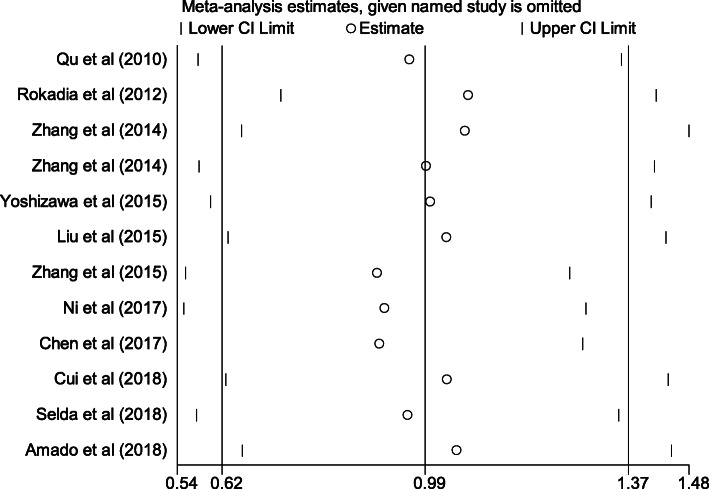
Sensitivity analysis on the associations between serum Cys C levels and COPD

To investigate potential correlations of serum Cys C levels with COPD, fifteen studies were enrolled for meta-analyses. We analyzed the heterogeneity of COPD vs control for the 12 studies, and the value of I^2^ value was 95.2% and *P* < 0.001. A heterogeneity was observed between these studies; therefore, the random-effects model was used for synthesis of the data. The pooled effect sizes indicated that the serum Cys C levels in patients with COPD were significantly higher than that in control (SMD = 0.99, 95%CI = 0.62–1.37, *P* < 0.001; Fig. 2). Moreover, we conducted a multivariate meta-regression analysis to explore the possible confounding factors leading to heterogeneity. The results showed that the year of publication, the case group, the proportion of cases and control, the ratio of men in cases and control, and the study quality as confounding factors had no significant effect on heterogeneity (*P* values were 0.589, 0.229, 0.480, 0.895 and 0.066). We further conducted a sensitivity analysis by successively extracting each study from this meta-analysis, which did not change the direction or statistical significance of SMD, indicating that this meta-analysis was stable (Fig. 3). In addition, Begg’s and Egger’s tests were used to evaluate publication bias, Begg’s test found no publication bias (*P* = 0.373), and publication bias was found by Egger’s tests (*P* = 0.007), therefore the trim and fill analysis was further performed and showed no further studies required [47].

### Subgroup meta-analysis

**Fig. 4:**
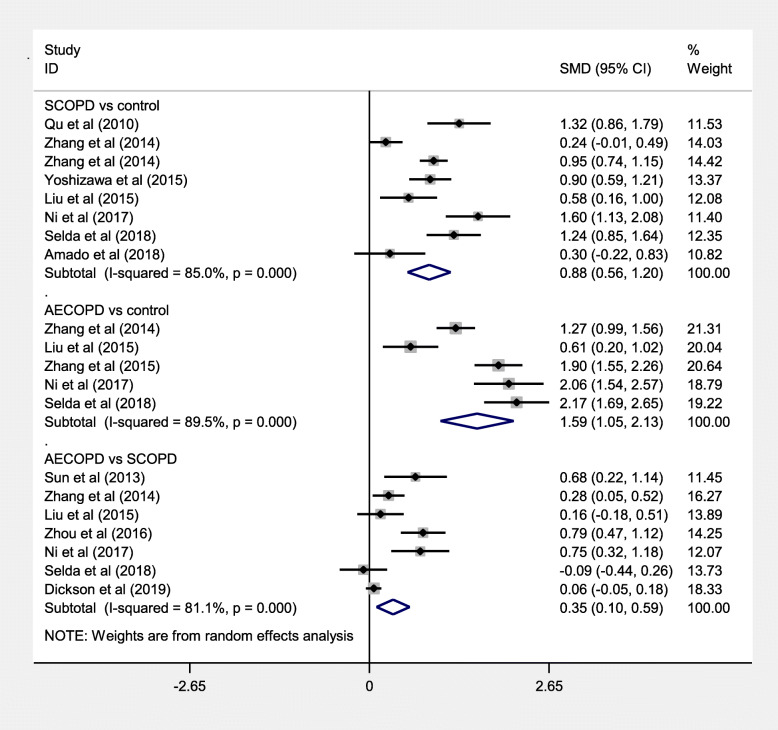
Meta-analysis of serum Cys C levels in AECOPD patients and SCOPD patients compared with control, and in AECOPD patients compared with SCOPD patients. Abbreviations: AECOPD, acute exacerbation COPD; SCOPD, stable COPD; SMD, standardized mean difference; CI, confidence interval

**Fig. 5:**
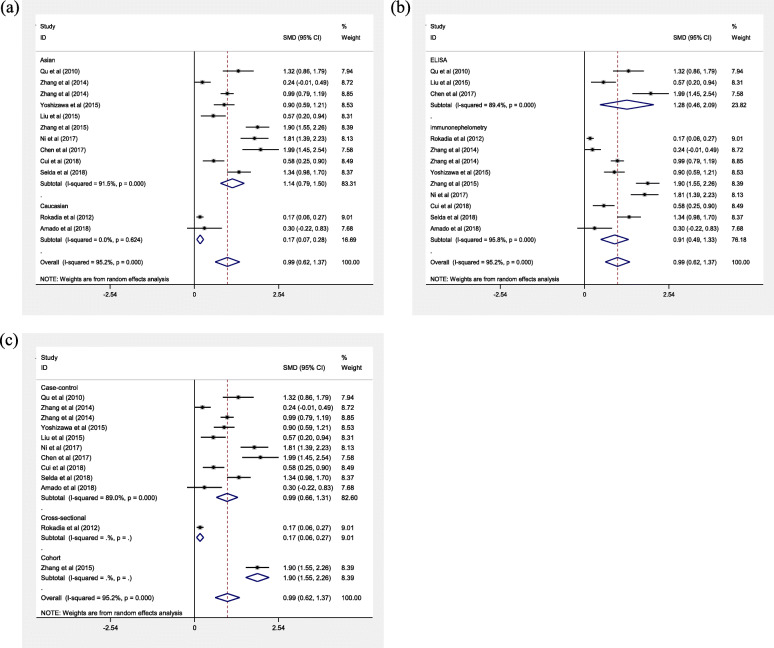
Meta-analysis of serum Cys C levels in COPD patients compared with controls. Note: Stratified analysis based on ethnicity **a**, assay methods **b**, study design **c**. Abbreviations: SMD, standardized mean difference; CI, confidence interval; ELISA, enzyme-linked immunosorbent assay

Due to the significant heterogeneity, we further performed subgroup analyses base on disease status, ethnicity, assay method, and study design. In the subgroup analysis of disease status (acute exacerbation COPD (AECOPD) vs control, stable COPD (SCOPD) vs control, AECOPD vs SCOPD) (Table 2), the meta-analysis results indicated that they were statistically different between AECOPD and controls (SMD = 1.59, 95%CI = 1.05–2.13, *P* < 0.001, Fig. 4), SCOPD and controls (SMD = 0.88, 95%CI = 0.56–1.20, *P* < 0.001), AECOPD and SCOPD (SMD = 0.35, 95%CI = 0.10–0.59, *P* = 0.005). The ethnicity-special subgroup analyses showed that COPD among the Caucasians had lower serum Cys C levels (SMD = 0.17, 95%CI = 0.07–0.28, *P* = 0.001, Fig. 5a) than subjects in COPD among the Asians (SMD = 1.14, 95%CI = 0.79–1.50, *P* < 0.001). Stratification by assay method showed that serum Cys C levels had significant associations with COPD among the immunonephelometry (SMD = 0.91, 95%CI = 0.49–1.33, *P* < 0.001, Fig. 5b) and ELISA (SMD = 1.28, 95%CI = 0.46–2.09, *P* = 0.002). In the subgroup analysis of study design, the results showed that COPD in the cohort study had higher serum Cys C levels (SMD = 1.90, 95%CI = 1.55–2.26, *P* < 0.001) than that in the case control studies (SMD = 0.99, 95%CI = 0.66–1.31, *P* < 0.001) and cross sectional study (SMD = 0.17, 95%CI = 0.06–0.27, *P* = 0.002, Fig. 5c). Due to the limited number of studies, sensitivity analysis and publication bias analysis were not performed in the subgroup analysis. The impact of heterogeneity slightly decreased for the Asians, immunonephelometry assay, ELISA assay, and case-control, but for the Caucasians, the impact of heterogeneity reduced to 0%. It indicated that ethnicity might be the source of heterogeneity.

**Table 2:** Characteristics of studies involving in comparison of the differences of serum Cys C levels

Author	Year	SCOPD			AECOPD			Control		
		n1	Mean1	SD1	n2	Mean2	SD2	n3	Mean3	SD3
Qu et al [37]	2010	70	1.76	0.62	/	/	/	30	0.98	0.51
Sun et al [39]	2013	30	0.95	0.23	54	1.12	0.26	/	/	/
Zhang et al [27]	2014	129	0.115	0.05	/	/	/	122	0.105	0.03
Zhang et al [29]	2014	299	1.03	0.21	93	1.09	0.22	151	0.84	0.18
Yoshizawa et al [40]	2015	108	1.2	0.4	/	/	/	73	0.9	0.2
Liu et al [41]	2015	60	0.1254	0.0309	72	0.1315	0.0425	36	0.1084	0.0267
Zhang et al [42]	2015	/	/	/	90	1.31	0.3	90	0.87	0.13
Zhou et al [43]	2016	60	0.94	0.22	108	1.13	0.25	/	/	/
Ni et al [44]	2017	45	0.1339	0.0154	45	0.1478	0.0212	45	0.1045	0.0209
Selda et al [31]	2018	68	1.01	0.56	58	0.97	0.29	50	0.47	0.13
Amado et al [28]	2018	65	0.97	0.32	/	/	/	18	0.88	0.2
Dickson et al [30]	2019	362	1.18	0.471	1637	1.21	0.47	/	/	/

*Abbreviations*: *COPD* Chronic obstructive pulmonary disease, *Cys C* Cystatin C, *AECOPD* acute exacerbation COPD, *SCOPD* stable COPD

### Meta-analysis of correlations between serum Cys C levels and clinical parameters

**Fig. 6:**
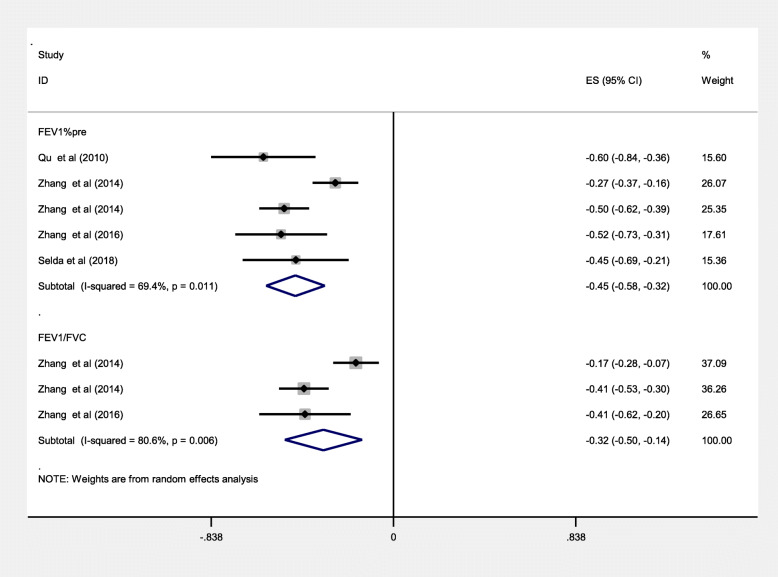
Meta-analysis of correlation coefficient between serum Cys C levels of COPD, FEV1%pre and FEV1/FVC. Abbreviations: CI, confidence interval; FEV1%pre, forced expiratory volume in 1 second as percentage of predicted volume; FEV1/FVC, forced expiratory volume in 1 s/forced vital capacity

We performed the correlation meta-analysis to investigate the relationships of serum Cys C levels with clinical parameters. The results showed that the meta-analysis indicated an inverse correlation between serum Cys C levels and FEV1%pre (Z = -0.45, 95%CI = -0.58--0.32, *P* = 0.011) or FEV1/FVC (Z = -0.32, 95%CI = -0.50--0.14, *P* = 0.006; Fig. 6).

## Discussion

This meta-analysis was designed to perform the potential relationship between serum Cys C levels and patients with COPD. The main results of the meta-analysis were as follows:1) the serum levels of Cys C in COPD patients were higher than that in the control; 2) serum Cys C levels in AECOPD patients and SCOPD patients were higher than those in the control group, and the serum Cys C levels in AECOPD patients were higher than that in stable COPD; 3) the serum Cys C levels were independent of ethnic, assay method and study design; 4) serum Cys C levels had a reverse correlation with FEV1%pre or FEV1/FVC.

Chronic obstructive pulmonary disease (COPD) is the most common Chronic respiratory disease, and the airflow limitations are associated with long-term chronic inflammatory responses of lung tissues to harmful gases and particles [7]. FEV1% and the frequency of exacerbations are common indicators for diagnosis and monitoring of treatment response [8]. However, due to the complicated examination of lung function, part of the patients cannot accept lung function as an index to evaluate the therapeutic effect. Therefore, it is necessary to look for sensitive and reliable markers related to COPD in simple sampling methods, which help clinicians diagnose COPD faster and evaluate its treatment effect more accurately.

The imbalance between protease and antiprotease is one of the important pathogenesis of COPD. Under the action of harmful factors, the protease system is stimulated and activated, which causes the synthesis and secretion of protease to be excessive, and the antiprotease system is destroyed and inactivated, which results that the synthesis or secretion of anti-protease is reduced. These together lead to an increase in the breakdown of matrix proteins in lung tissue, which promotes the development and progression of COPD [2].

Cathepsin is a family of cysteine proteases, and cathepsin B, H, L, and S are involved in the pathogenesis of COPD [38]. Cys C is a cysteine protease inhibitor, which is the most extensive and effective inhibitor of cathepsin involved in lung tissue destruction [48]. On the one hand, hypoxemia can damage inflammatory cells in the body and releases a large amount of Cys C, which leads to elevated levels of serum Cys C in patients with COPD [49]. On the other hand, hypoxia causes inflammatory cells such as macrophages to release the cytokines with protease activity, which results in the destruction of elastin and leads to emphysema. However, the increasing and activating of protease stimulates the body to synthesize more anti-protease, which will fight against the decomposition and destruction of protease to tissues. Therefore, the levels of Cys C can predict cathepsin activity indirectly and reliably [26]. Finally, when the body is in a state of hypoxia, the renin-angiotensin-aldosterone system (RAAS) and the sympathetic nervous system will be activated, which leads to the contraction of systemic small blood vessels, the decreasing of glomerular blood flow into the aorta, the reducing of effective glomerular filtration rate, and ultimately increasing serum Cys C levels.

This meta-analysis indicated that serum Cys C levels were higher in COPD. Nakajima et al. [26] reported that serum levels of Cys C were significantly higher in COPD patients than control. Previous studies showed that inflammatory parameters such as interleukin-6, resistin, tumor necrosis factor, and CRP were significantly correlated with Cys C, suggesting that the elevation of Cys C was secondary to the inflammatory processes in the lung [31, 38, 50]. These all above cytokines have been found to be closely related to COPD, which indicated that elevated Cys C levels might be associated with pulmonary inflammation.

Consistent with previous studies, we found that the elevated levels of Cys C were significantly correlated with disease status and lung function. Hu et al. [24] found that the elevated Cys C levels was an important and independent risk factor of increased mortality in a hospital setting during COPD exacerbation, Formiga et al. [51] indicated that inspiratory muscle function was reduced with greater degrees of inflammation in COPD as expressed higher levels of Cys C. Combining with the results of previous studies and this meta-analysis, we thought that the elevated levels of serum Cys C might indicate the exacerbation and progression of COPD.

In this meta-analysis, we noted that there was great heterogeneity in the included studies. Moreover, we performed Egger’s test, which revealed that there was significant evidence of publication bias in the included studies. Although we used the random-effect model when we found heterogeneity, this increases the probability of type I error. We carried out a sensitivity analysis to assess the stability of this meta-analysis. Sequential removal of each study did not alter the conclusions, suggesting that these results were reliable. We also performed meta-regression analysis and subgroup analysis to perform the possible sources of heterogeneity and reduce the occurrence of type I errors.

Before explaining our results, several limitations of this meta-analysis should be pointed out. First, although some confounding factors might influence the results of the meta-analysis, we did not conduct a subgroup analysis of smoking status due to insufficient original data, the lack of original data also might limit sufficient statistical power to evaluate the potential effects of Cys C levels on the development of COPD. Second, although we tried to gather the original data from the study authors, few authors responded to the data request. Hence, we adopted accepted methods to extract and synthesize data. Third, the concentration of serum Cys C is affected by a variety of diseases, and a part of these diseases are associated with COPD. Previous studies have indicated that Cys C is strongly associated with renal function [52, 53], chronic hypoxia could also lead to renal dysfunction in patients with COPD [54]. Although patients with kidney disease were excluded when we included the literature, the renal dysfunction caused by COPD might affect the concentration of serum Cys C, which may have some influence on the results. Fourth, the sample sizes in most cited studies were small, and methodologic design may be flawed in smaller studies, the conclusions of smaller studies might be impacted by confounding factors, which might have an impact on our results. Fifth, the probability of death due to COPD is much higher in Asian, particularly China, than western countries [55, 56], and Caucasians with COPD have lower serum Cys C level compared with Asians COPD patients [28, 30, 38, 42]. Although we had done a review of the available published studies, most studies included were from Asia, therefore the promotion of conclusions had some limitations. Sixth, many studies have indicated that elevated Cys C levels may be associated with pulmonary inflammation. However, due to the insufficient data, we did not perform the causal mediation analysis to explore whether serum Cys C was an inflammatory mediator in the development of COPD [57], which made the extension of the results limited. Finally, only published studies were retrieved in this meta-analysis and possible publication bias might exist, and Egger’s test found significant evidence of publication bias. Therefore, more keywords should be used to retrieve more studies for further evaluating the relationship between Cys C levels and COPD.

## Conclusion

Conclusively, the current meta-analysis suggests that, serum Cys C levels are higher in patients with COPD (both stable COPD and AECOPD) compared to controls. Serum Cys C levels are reversely correlated with FEV1%pre or FEV1/FVC. The results provided an improved understanding of the roles of Cys C in COPD development and progression. Further large-scale, unified and well-designed studies are needed to explore the relationship between Cys C and COPD, COPD exacerbation.

## Data Availability

All data generated or analyzed during this study are included in this published article.

## Abbreviations

COPD: Chronic obstructive pulmonary disease
Cys C: Cystatin C
GFR: glomerular filtration rate
SMD: Standardized mean difference
CNKI: China National Knowledge Infrastructure
CI: Confidence interval
NOS: Newcastle-Ottawa scale
AECOPD: Acute exacerbation COPD
SCOPD: Stable COPD
FEV1%pre: Forced expiratory volume in 1second as percentage of predicted volume
FEV1/FVC: Forced expiratory volume in 1 s/forced vital capacity.
